# Uncertainty Analysis of Mobile Phone Use and Its Effect on Cognitive Function: The Application of Monte Carlo Simulation in a Cohort of Australian Primary School Children

**DOI:** 10.3390/ijerph16132428

**Published:** 2019-07-08

**Authors:** Christopher Brzozek, Kurt K. Benke, Berihun M. Zeleke, Rodney J. Croft, Anna Dalecki, Christina Dimitriadis, Jordy Kaufman, Malcolm R. Sim, Michael J. Abramson, Geza Benke

**Affiliations:** 1 Centre for Population Health Research on Electromagnetic Energy (PRESEE), School of Public Health and Preventive Medicine, Monash University, Melbourne, VIC 3004, Australia; 2 School of Engineering, University of Melbourne, Parkville, VIC 3010, Australia; 3 AgriBio, Centre for AgriBioscience, Bundoora, VIC 3083, Australia; 4 Australian Centre for Electromagnetic Bioeffects Research (ACEBR), Illawarra Health and Medical Research Institute, School of Psychology, University of Wollongong, Northfields Ave, Wollongong, NSW 2522, Australia; 5 School of Health Sciences, Swinburne University of Technology, Hawthorn, VIC 3122, Australia

**Keywords:** radiofrequency electromagnetic fields, mobile phones, uncertainty analysis, Monte Carlo simulation, cognitive function

## Abstract

Previous epidemiological studies on health effects of radiation exposure from mobile phones have produced inconsistent results. This may be due to experimental difficulties and various sources of uncertainty, such as statistical variability, measurement errors, and model uncertainty. An analytical technique known as the Monte Carlo simulation provides an additional approach to analysis by addressing uncertainty in model inputs using error probability distributions, rather than point-source data. The aim of this investigation was to demonstrate using Monte Carlo simulation of data from the ExPOSURE (Examination of Psychological Outcomes in Students using Radiofrequency dEvices) study to quantify uncertainty in the output of the model. Data were collected twice, approximately one year apart (between 2011 and 2013) for 412 primary school participants in Australia. Monte Carlo simulation was used to estimate output uncertainty in the model due to uncertainties in the call exposure data. Multiple linear regression models evaluated associations between mobile phone calls with cognitive function and found weak evidence of an association. Similar to previous longitudinal analysis, associations were found for the Go/No Go and Groton maze learning tasks, and a Stroop time ratio. However, with the introduction of uncertainty analysis, the results were closer to the null hypothesis.

## 1. Introduction

Mobile (cellular) phones have become very common in social contexts and are used for communications, internet browsing, as global positioning systems, calendars, and other program applications (apps). Children and adolescents often own mobile phones and are being exposed to the radiofrequency electromagnetic fields (RF-EMF) at younger ages [1,2,3,4]. One emerging area of research interest is the possible health effects that mobile phone RF-EMF may have on the developing cognitive function of children and adolescents. The World Health Organization (WHO) identified prospective cohort studies of children and adolescents with outcomes, including behavioral and neurological disorders as a high-priority research need [5]. Despite this, the scientific consensus on the health risks of mobile phones on children and adolescents is still vague and inconclusive. Specifically, previous epidemiological studies investigating the effects of RF-EMF on adolescent cognitive functions have found contradictory results [4,6,7,8,9,10,11]. Recent research has highlighted experimental difficulties and various uncertainties that can affect the results of studies, including statistical variability, measurement error, and model uncertainty, all of which may be responsible for the inconsistent results within and between studies [12].

The application of uncertainty analysis is strongly recommended in order to gain more value from epidemiological studies and to aid in risk assessment [13,14]. This is especially true for RF-EMF research, which has been the subject of great variability in results. A recent workshop on risk assessment stressed the need for much greater research into uncertainty analysis of epidemiological data [15]. The workshop cited the hierarchy proposed by the National Research Council (USA 2009), which classified risk as a probability of an adverse event, analogous to the *p*-value [16]. This was only a point estimate and represented the lowest ‘tier’ of four levels in uncertainty analysis and should be regarded as the default level. A risk probability is not equivalent to an uncertainty interval, which is the top tier providing outcome distributions for bounding estimates such as the confidence interval.

The effect of uncertainty in data can be illustrated conceptually by the difference between two population means. Consider the z-test, where the statistical significance increases with the ratio Z = (µ_A_ − µ_B_) / (σ²_A_ + σ²_B_). In the deterministic case with a single measurement, the denominator is a constant, as there is no variance. This assumes error-free data collection. In the statistical case, the denominator represents the effect of experimental replications. With the addition of epistemic uncertainty (lack of information), the sum of variances may also include measurement error due to instrumentation, variability in expert opinion, data entry errors, etc. These additional errors lower the z-value and therefore decrease the statistical significance of the difference between the treatment means. When enough uncertainties are included, or their magnitudes are significant, the difference between the two means may no longer be statistically significant. This can affect the acceptance or rejection of a null hypothesis and emphasizes the need for measurement error to be addressed, preferably by quantitative methods such as probability distributions.

An uncommon approach in the epidemiological literature is the use of Monte Carlo simulation, which can be used to quantify measurement errors and their propagation through the mathematical structure of a model. This approach to uncertainty analysis and its propagation can be used to combine statistical variability with epistemic uncertainties [12]. With respect to statistical variability, there will always be residual errors, although they can be characterized by probability distributions for the purpose of Monte Carlo simulation [17].

Monte Carlo simulation is a powerful technique for analyzing statistical variability and its propagation in both single and complex coupled models, and has been used successfully in many other disciplines [18]. A primary aim of this paper was to apply the use of Monte Carlo simulation methodology in the ExPOSURE study for the quantification of uncertainty in the output of the model. This study aimed to investigate the effects of mobile phone use with cognitive function and to address concerns over model uncertainty by updating the covariate structure used in the model.

## 2. Methods

### 2.1. Study Design

The detailed design and methods in the ExPOSURE study have been described previously [7,9]. In brief, the ExPOSURE study was a prospective cohort study of fourth year Australian primary school children in Melbourne and Wollongong between 2011 and 2013. Initially, 619 students were recruited at baseline, and longitudinal data were collected for 412 students from a representative sample of 36 state, private, and Catholic schools. The parents or guardians of the children from the classes selected by the school were then sent plain language information packs detailing the study and consent forms. Students were excluded if they had a known cognitive disorder (such as Attention Deficit Hyperactivity Disorder).

Participants were examined at baseline and follow-up, and each examination required the parents/guardians to complete a questionnaire, which gathered information on their children’s mobile phone use as well as sociodemographic data. Students completed a shorter questionnaire regarding mobile phone ownership, handedness, and use of other electronic devices. They also completed a computerized cognitive function battery and the Stroop color-word test. Principals, teachers, parents/guardians, and students provided written informed consent. The study was approved by the Victorian Department of Education and Early Childhood Development, the New South Wales Department of Education and Communities, the Catholic Education Offices of Victoria and New South Wales, and the Monash University and University of Wollongong Human Research Ethics Committees.

### 2.2. Exposure Assessment

Mobile phone use was assessed via a validated questionnaire derived from the INTERPHONE study [19]. The parents or guardians completed this questionnaire, which included questions on the child’s ownership and use of mobile phones. This included the average number and duration of voice calls made and received per week on mobile phones. Sociodemographic data such as age, sex, ethnicity (country of birth), and residential postcode were also gathered. The children completed a shorter questionnaire, which included questions on mobile phone ownership, handedness, physical activity (times per week), and the time spent on the internet and computerized gaming consoles per week.

### 2.3. Outcome Assessment

The validated computerized psychometric test battery CogHealth (CogState, Melbourne, Australia, 2005) and the Stroop color-word test were used to assess several cognitive function domains [20,21,22,23,24]. CogHealth evaluated the response time (ms) and accuracy (%) of simple reaction time with the detection test, choice reaction time with the identification task, working memory with the one-back task, and visual recognition and memory with the one card learning task and response inhibition with the Go/No Go task. Spatial and executive ability were also assessed by CogHealth via the total number of errors recorded while completing the Groton maze learning task. Further details of these specific tasks are discussed elsewhere [6]. The Stroop test consists of four tasks that measure the participant’s vulnerability to interference caused by concurrent automatic processing of color words and color hue. Further details of this test and the component tasks can be found in the literature [6,9,20].

### 2.4. Statistical Analysis

Mobile phone use was analyzed as the average number of calls made and received per week over the study period using the self-reported number of calls at baseline and follow-up. In general, the self-reported mobile phone calls at baseline served as the lower limit, as most participants’ phone usage increased over time, and the follow-up exposure data served as the upper limit. There was a small proportion of participants whose mobile phone use decreased at follow-up. Therefore, for these participants, the baseline exposure was the upper limit and the follow-up exposure was the lower limit. Call duration was not included, as previous research has shown recalled call duration to be inaccurate [25,26]. SMS data was also not included, as RF-EMF exposure to the head while sending or receiving an SMS text was minimal. Monte Carlo simulation was used to address uncertainty in the inputs [14,18,27,28,29].

The Monte Carlo simulation process was implemented as follows. The traditional point estimate for a call was replaced by an interval, with lower and upper bounds to be used as the parameters for the uniform probability distribution to characterize input uncertainty. The probability distribution for each participant was sampled, and the mean of all participants was then computed. This process was repeated for 1000 trials to produce the output probability distribution. From this distribution, the mean and confidence interval were computed. The procedure is analogous to quantitative risk analysis for estimation of average risk using a group of experts [14].

The random number generator used in the simulation was the Mersenne Twister algorithm and convergence was accelerated using Latin Hypercube sampling, rather than simple random sampling. Results were produced using a commercial software package for Monte Carlo simulation (viz. @RISK ver 7.5, Palisade Corporation, Ithaca, NY, USA). This process was applied to participants using mobile phones in order to determine their average weekly exposure over the study period. Based on the probability distributions from the Monte Carlo simulation results, students who were more likely to have averaged less than or equal to two calls per week were in the low-exposure group, while those who averaged over two calls a week were in the higher exposure group. Participants who reported making no calls formed the control group. This process was also repeated for the mean number of calls for the exposed participants.

The mean response times for cognitive parameters were log transformed while accuracy was arcsine transformed as recommended by CogState Research for analysis. The Stroop color-word test response times and error rates were analyzed by comparing the results from Form B with Form A, and the results of Form D with Form C. Multiple linear regression models were fitted to each of these cognitive parameter outcomes for mobile phone use. Robust standard errors were used to allow for clustering of students within schools. Age, sex, country of birth, physical activity (times active per week), socioeconomic status (in quintiles based on postcodes and the Socioeconomic Index for Areas), time between examinations, baseline cognitive function score, and time spent console gaming per week (quintiles) were identified and fitted as covariates based on a previously developed Directed Acyclic Graph (DAG) presented in the Supplementary Materials [12]. An interaction between sex and computerized console gaming found in previous research was included in the multiple linear regression models [30]. Data analysis was conducted with STATA version 13 (StataCorp, College Station, TX, USA). A *p*-value less than or equal to 0.05 was considered statistically significant.

## 3. Results

### 3.1. Descriptive Data

Initially, 1189 students were invited to take part in the study. Of those, 619 students agreed to participate (52% participation rate), with 412 students participating in both baseline and follow-up (66.5% follow-up rate). Students with a known cognitive disorder (*n* = 8) were excluded from the analysis. At baseline, the participants’ mean age was 9.9 (SD = 0.5) years with 329 female participants (53%). At follow-up, the mean age had increased to 11 (SD = 0.5) years with 227 female participants (55%). The proportion of parents/guardians reporting mobile phone ownership or use increased from 31% at baseline to 43.3% at follow-up. However, the proportion of participant self-reported mobile phone ownership or use also increased and remained higher than the reported parent/guardian figures, with 57.3% at baseline to 67.9% at follow-up. 

**Figure 1:**
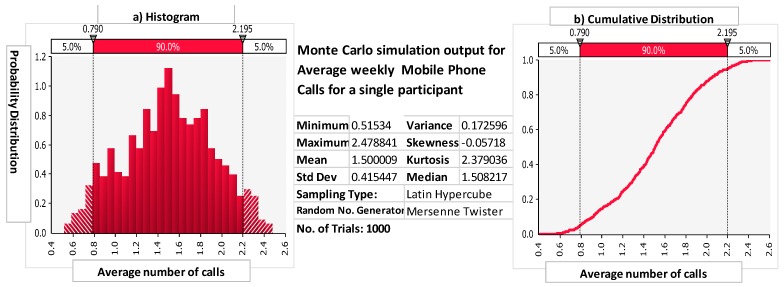
(**a**) Monte Carlo simulation for the average number of mobile phone calls after 1000 trials for an individual participant. Depicted is the histogram, plotting the probability distribution (*y*-axis) vs. average weekly mobile phone calls (*x*-axis) after *n* = 1000 trials, and (**b**) cumulative distribution showing risk probability vs. average weekly mobile calls.

Figure 1 is an example of the Monte Carlo simulation output for average weekly mobile phone calls made by a single participant. The results in Figure 1 show that for this participant, there was greater than an 80% probability that he/she averaged less than two mobile phone calls over the study period. Therefore, this participant was categorized in the low-exposure group for mobile phone exposure.

**Figure 2:**
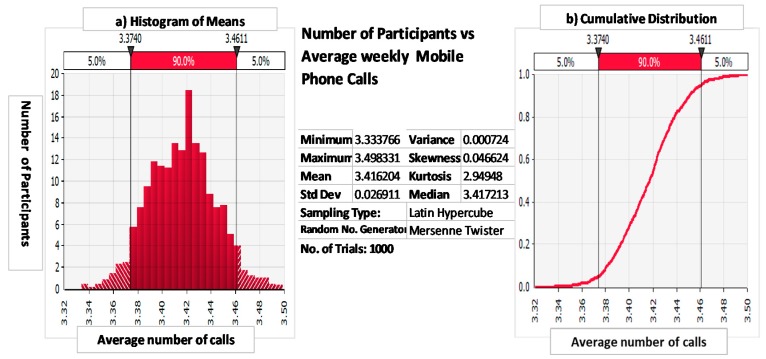
(**a**) Monte Carlo simulation for mean number of calls for trial with *n* = 163 participants. Depicted is the histogram, plotting number of participants (*y*-axis) vs. average weekly mobile phone calls (*x*-axis) after *n* = 1000 trials, and (**b**) cumulative distribution showing risk probability vs. average weekly mobile calls, e.g., *x* ≤ 3.46 for *p* = 0. Uncertainty is defined as the confidence interval for the means for a specified level of statistical significance.

Table 1 shows descriptive statistics for the exposure variable used in the analysis. These were determined based on the individual Monte Carlo simulation outputs for each participant as demonstrated in Figure On average, 3.42 mobile phone calls were made per week, as shown in Figure 2.

### 3.2. Cognitive Function

Performance on CogHealth tasks improved from baseline to follow-up, with an overall reduction in response times found for all tasks. Accuracy improved or remained steady across all tasks with improvement particularly seen at the 25th percentile. The total number of errors made in the Groton maze learning task also fell. The Stroop response time ratio for Form (B-A)/A increased indicating participants at follow up experienced greater interference, while for Form (D-C)/C the time ratio decreased indicating less interference. Table 2 shows descriptive statistics for CogHealth tasks and Stroop time ratios.

### 3.3. Associations between Mobile Phone Use and Cognitive Outcomes

Table 3 shows the results of the linear regression models for average weekly mobile phone calls over the study period with transformed cognitive outcomes. Low and higher exposure groups, as determined via the Monte Carlo simulations, were compared with the no use reference category. Statistically significant associations were found in both the low and higher mobile phone call groups for the Go/No Go response inhibition task (low: −0.023, 95% CI: −0.042, −0.004; higher −0.024, 95% CI: −0.046, −0.001). The low mobile phone call group was found to make more errors while completing the Groton maze learning task (4.53, 95% CI: 1.10, 7.96).However, no association was seen in the higher mobile phone call group. A significant association was also found in the low mobile phone call group for Stroop time ratio AB, as they were significantly slower in completing Form B compared to Form A (0.034 95% CI: 0.001, 0.068). The results indicate that there was greater interference between the two forms in this group.

## 4. Discussion

Overall, the results of this study show limited evidence of an association between mobile phone use and cognitive function outcomes. The previous longitudinal analysis, which investigated whether an increase in mobile phone use at follow-up from baseline was associated with cognitive function changes, found similar significant associations. However, these associations were stronger than in the current analysis (Go/No Go task −0.030, 95% CI: −0.054, −0.006; Groton maze learning task 6.22, 95% CI: 2.13, 10.31; Stroop time ratio AB 0.056 95% CI: 0.021, 0.090) [7]. However, with input uncertainty addressed via Monte Carlo simulation, the results of the current analysis were closer to the null. The shorter response time associations and poorer inhibitory function (poorer Stroop time ratio (B-A)/A) findings are consistent with the previous research of the MoRPhEUS study, which also found shorter response times for simple and associative learning tasks [6]. Yet, the MoRPhEUS study also found less accurate results for working memory and associative learning, which this study did not [6].

Negative associations between memory performance and cumulative duration of wireless phone use and higher RF-EMF doses have also been found in the HERMES study [11]. The HERMES results were more strongly associated with RF-EMF doses suggesting RF-EMF exposure affects memory performance. However, other studies have found that learned smartphone-related behaviors, rather than RF-EMF doses, are detrimental to memory performance [31]. Those with a greater frequency of media multitasking have been shown to have reduced working memory capability [32]. Sparrow et al. reported that participants having lower rates of information recall and instead enhanced recall for where to access information, and concluded that human memory was adapting to the latest technologies [33]. Therefore, it is difficult to determine whether these associations are with mobile phone behaviors or RF-EMF doses.

In this study, mobile phone calls were associated with a lower accuracy (by increasing the number of errors) of the Groton maze learning task. However, as with the Stroop task time ratio. this was only statistically significant in the low mobile phone call group and not found in the higher call group. Therefore, caution is recommended when interpreting these results. Results of the response inhibition Go/No Go task were also inconsistent as the longitudinal analysis found contrasting results to the cross-sectional baseline analysis. Despite both the low and higher mobile phone call groups showing faster response times when compared to the ‘no use’ reference category, the baseline analysis found mobile phone use to be associated with slower response times [9]. Consequently, we consider that this is most likely a chance finding.

### Strengths and Limitations

A novel feature of the investigation, not previously observed by the authors in other studies in this area, was the introduction of Monte Carlo simulation to quantify uncertainty in the output of the model through computing error propagation in the input data. This approach has potential for use and extension in future studies. Other strengths of this study were as follows: The use of stratified sampling across all three school sectors, namely state, private, and Catholic schools. The increased statistical confidence due to the relatively large sample size included. The comprehensive assessment of seven cognitive tasks via the well-validated and age-appropriate cognitive test batteries CogHealth and the Stroop color-word test [21,34]. Another strength of the study was that epistemic sources of uncertainty were addressed. A DAG was used to display the structure of the associations between variables of interest and identify confounders and effect modifiers, thus reducing context uncertainty. This uncertainty was further reduced with the finding of an interaction between sex and console gaming, which was included in the models [30].

The primary limitation of this study was the self-reported, rather than measured, RF radiation exposure ascertainment. It has been revealed in past studies that uncertainties can be introduced by self-reporting, particularly call duration, which is less accurate than objectively recorded data [25,35,36]. Therefore, despite call duration being important to determining a person’s RF-EMR exposure, it was not included in the exposure assessment and the average number of calls per week was used instead. In this study, the questionnaire used to gather exposure assessment data was based on a previously validated questionnaire [19]. Other limitations are that despite model adjustments and the inclusion of numerous potential confounders, it cannot be ruled out that an important confounder was missed.

Despite the use of Monte Carlo simulation to address input uncertainties, it is further recommended that future research prioritizes objectively recorded exposure data to decrease epistemic uncertainty due to measurement errors. Billing records are a common alternative, but have significant short comings despite providing accurate data on the number of calls made and call duration. They lack information on the use of loud speaker, laterality, signal strength, and calls made through apps such as WhatsApp, Messenger, etc., which are important when assessing RF-EMR exposure [12]. With billing records becoming increasingly harder to obtain in many countries, alternative exposure assessment techniques, such as the use of mobile phone apps, will need to be assessed. XmobiSense and QuantaMonitor™ are two possible apps that could be considered [37].

## 5. Conclusions

There was little evidence to suggest any significant association between the use of mobile phones and cognitive function in school students. As with the previous longitudinal analysis, associations were found between mobile phone use and the Go/No Go task, the Groton maze learning task, and a Stroop time ratio. However, with the introduction of uncertainty analysis techniques, these results were closer to the null hypothesis. A novel feature of the study was to raise the issue of uncertainty analysis, including investigation of a number of epistemic uncertainties in addition to statistical variability. The uncertainties identified included model uncertainty as well as measurement error, which was addressed by replacing point source estimates of calls with error probability distributions. Monte Carlo simulation was used to evaluate error propagation from inputs to outputs of the exposure model. The confidence intervals computed in this manner are more reasonable estimates of model output uncertainty in contrast to only using point source estimates. This stochastic approach adds value to traditional statistical analysis and is recommended for further investigation and evaluation in future studies.

## Figures and Tables

**Table 1:** Descriptive statistics for mobile phone use in average number of calls made and received per week over a year, as reported by the participant’s parents/guardians.

Exposure Mins/Week	Median (25th, 75th Percentile)		
	None	Low	Higher
Mobile Phone calls	0 (0, 0) *n* = 219	1 (0.5, 1.5) *n* = 84	5 (3, 7.5) *n* = 79

Low ≤ 2 calls per week on average throughout the year Higher > 2 calls on average over a year.

**Table 2:** Descriptive statistics for cognitive function outcomes as assessed by CogHealth and Stroop color-word test time ratios.

Variable	Metric	Baseline ^1^	Follow-Up ^1^
Detection Task (Simple reaction time)	Response time (ms^2^)	348 (304, 415)	317 (287, 356)
	*n* = 397		
	Accuracy (%)	97 (90, 100)	97 (95, 100)
	*n* = 402		
Identification Task (Choice reaction time)	Response time (ms^2^)	596 (528, 683)	542 (484, 602)
	*n* = 401		
	Accuracy (%)	94 (86, 97)	94 (88, 97)
	*n* = 402		
One-back memory task (Working memory)	Response time (ms^2^)	972 (800, 1125)	865 (712, 1011)
	*n* = 401		
	Accuracy (%)	89 (76, 94)	91 (84, 97)
	*n* = 402		
One card learning task (Visual recognition memory and attention)	Response time (ms^2^)	1103 (885, 1324)	1033 (875, 1225)
	*n* = 402		
	Accuracy (%)	59 (49, 65)	64 (55, 70)
	*n* = 402		
Go/No Go task (Response inhibition)	Response time (ms^2^)	632 (556, 720)	578 (507, 663)
	*n* = 399		
	Accuracy (%)	96 (94, 100)	98 (96, 100)
	*n* = 400		
Groton maze learning task (Spatial and executive ability)	Total number of errors	69 (54, 84)	56 (47, 69)
	*n* = 402		
Stroop Form (B-A)/A	Time Ratio	0.09 (0.02, 0.17)	0.11 (0.04, 0.21)
Stroop Form (D-C)/C	Time Ratio	0.69 (0.54, 0.90)	0.65 (0.51, 0.83)

^1^ Median and Interquartile range; ^2^ Milliseconds.

**Table 3:** Difference in adjusted means for cognitive outcome scores by ‘annual cumulative mobile exposure’ model.

Test	Parameter	‘Low’ Group		‘Higher’ Group	
		Estimate	95% CI	Estimate	95% CI
Detection Task (Simple reaction time)	Speed ^1^	−0.015	−0.035, 0.005	−0.0002	−0.019, 0.019
	Accuracy ^2^	−0.002	−0.048, 0.045	0.002	−0.036, 0.04
Identification Task (Choice reaction time)	Speed ^1^	0.001	−0.020, 0.023	−0.010	−0.026, 0.005
	Accuracy ^2^	0.006	−0.037, 0.050	0.028	−0.018, 0.074
One-back memory task (Working memory)	Speed ^1^	0.013	−0.011, 0.037	−0.0001	−0.022, 0.022
	Accuracy ^2^	0.001	−0.048, 0.051	−0.030	−0.094, 0.035
One card learning task (Visual recognition memory and attention)	Speed ^1^	0.001	−0.023, 0.026	0.009	−0.023, 0.042
	Accuracy ^2^	0.005	−0.022, 0.032	−0.016	−0.047, 0.016
Go/No Go task (Response inhibition)	Speed ^1^	−0.023 *	−0.042, −0.004	−0.024 *	−0.046, −0.001
	Accuracy ^2^	−0.014	−0.044, 0.016	−0.002	−0.044, 0.040
Groton maze learning task (Spatial and executive ability)	Total number of errors	4.53 *	1.10, 7.96	1.77	−2.40, 5.94
Stroop time ratio AB	Response time ratio (B-A)/A	0.034 *	0.001, 0.068	0.004	−0.024, 0.032
Stroop time ratio CD	Response time ratio (D-C)/C	−0.036	−0.117, 0.045	−0.042	−0.096, 0.012

Reference group: Students who did not use or own a mobile phone. Regression coefficients adjusted for age, sex, country of birth, physical activity, socioeconomic status, time between examinations, baseline cognitive function score, and time spent console gaming per week, with an interaction between sex and computerized console gaming. ^1^ Base 10 log transformed data; ^2^ Arcsine transformed data; * Statistically significant results.

## Supplementary Materials

The following are available online at https://www.mdpi.com/1660-4601/16/13/2428/s1, Figure S1: Directed acyclic graph of covariate structure. Copied with permission of authors from Brzozek et al. [12].
