# Supplementary material for: Uncertainty Analysis of Mobile Phone Use and Its Effect on Cognitive Function: The Application of Monte Carlo Simulation in a Cohort of Australian Primary School Children
Source: Int J Environ Res Public Health. 2019 Jul 8;16(13):2428. doi: 10.3390/ijerph16132428 (PMC6651811; doi:10.3390/ijerph16132428)
Supplement: Supplementary file 1 [file ijerph-16-02428-s001.pdf]

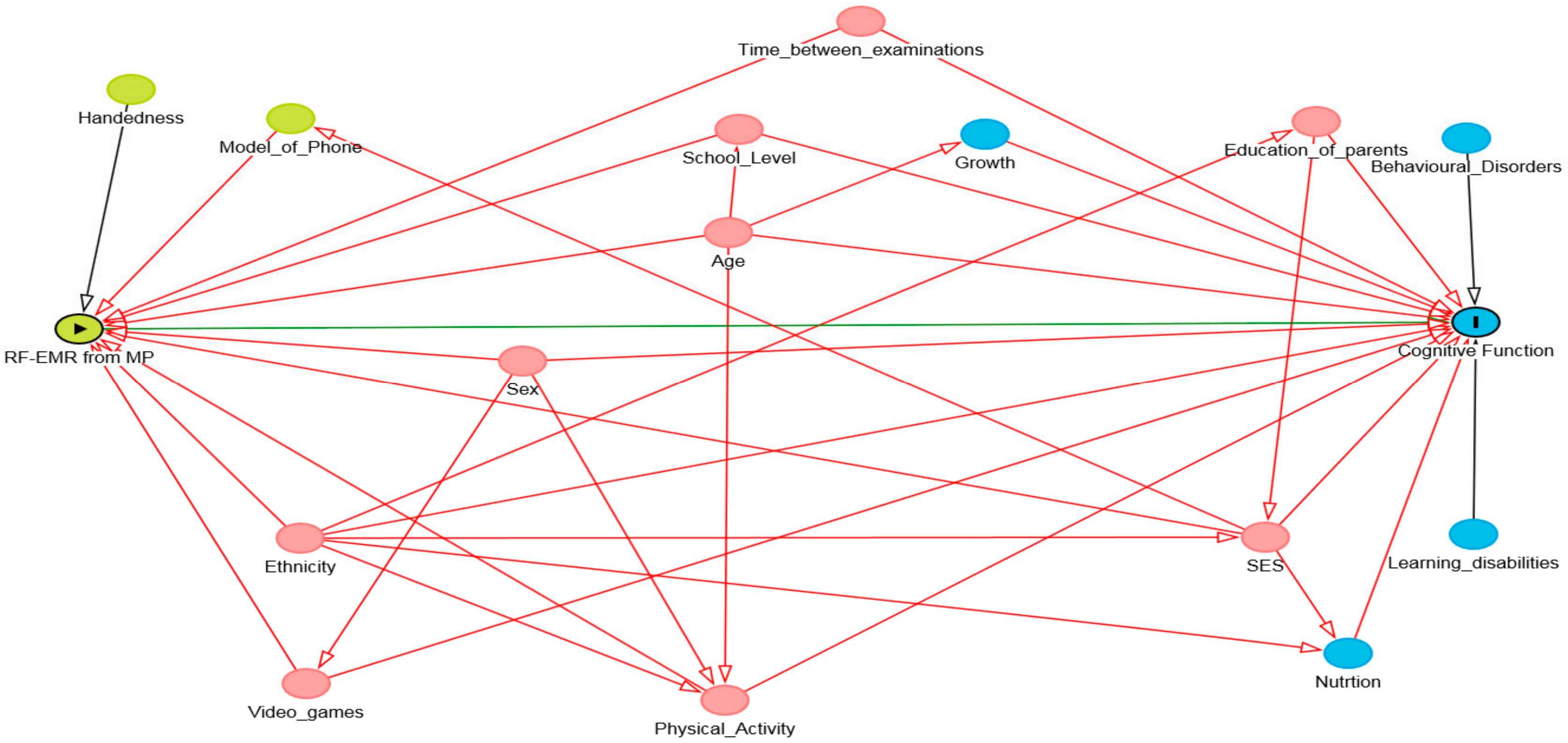

Supplementary Figure S1. Directed acyclic graph used to determine covariate structure. Copied with permission of authors from Brzozek et al. [12]
